# Impact of Preterm Birth on Long-Term Cardiac Function: A Comprehensive Echocardiographic Study in School-Aged Children

**DOI:** 10.3390/medicina61040573

**Published:** 2025-03-23

**Authors:** Nidai Dalokay, Ayse Sulu, Pelin Kosger, Tugba Barsan Kaya, Birsen Ucar

**Affiliations:** 1 Department of Pediatrics, Faculty of Medicine, Eskişehir Osmangazi University, Eskisehir 26040, Turkey; nidaidalokay@gmail.com; 2 Department of Pediatric Cardiology, Faculty of Medicine, Eskisehir Osmangazi University, Eskisehir 26040, Turkey; pkosger@ogu.edu.tr (P.K.); bucar@ogu.edu.tr (B.U.); 3 Department of Pediatric Cardiology, Faculty of Medicine, Gaziantep University, Gaziantep 27310, Turkey; 4 Department of Neonatology, Faculty of Medicine, Eskişehir Osmangazi University, Eskisehir 26040, Turkey; tugba.barsankaya@ogu.edu.tr

**Keywords:** prematurity, strain echocardiography, tissue doppler echocardiography

## Abstract

*Background and Objectives*: This study aimed to evaluate the cardiac functions of 7- to 11-year-old children with a history of preterm birth using echocardiography and to assess the relationship between these functions and neonatal factors. *Materials and Methods*: A total of 64 children were included in the study, consisting of 32 children aged 7 to 11 years with a history of preterm birth and 32 age- and gender-matched term birth controls. *Results*: While no significant differences were detected between the preterm and term birth groups regarding age, height, and body weight, echocardiographic data revealed higher values of mitral E, mitral A, and tricuspid A by pulse wave Doppler, as well as septal E by tissue Doppler, in the preterm group compared to the term birth group (*p* < 0.05). Additionally, the left ventricular global longitudinal peak strain, right ventricular free wall and right ventricular 4-chamber strain, IVRT, MPI, MAPSE, and LVESV values were lower in the preterm group than in the term birth group (*p* < 0.05). No significant differences were detected in circumferential strain measurements. Right ventricular strain measurements were significantly lower in the preterm group (*p* = 0.001). *Conclusions*: While conventional echocardiographic examinations did not reveal obvious pathological findings in school-age children with a history of preterm birth, further echocardiographic assessments demonstrated differences compared to term birth controls, particularly in diastolic functions and right and left ventricular longitudinal strain measurements.

## 1. Introduction

Preterm birth accounts for more than 9.9% of births worldwide and is associated with an increased risk of cardiovascular disease in the long term [1,2]. While extensive studies have been conducted on long-term neurodevelopmental issues, the effects of prematurity on other systems remain poorly understood. It has been shown that individuals born preterm have an increased risk of cardiovascular conditions such as hypertension and ischemic heart disease in the long term [3]. Each year, the number of preterm births and the percentage of surviving individuals continue to increase, leading to cardiovascular mortality and morbidity in adulthood. Preterm birth is a risk factor for heart failure during childhood and young adulthood [4]. During late fetal life, especially in the third trimester, terminal differentiation occurs in cardiomyocytes. Since the heart loses its proliferative capacity (hyperplasia) immediately after birth, cardiomyocyte maturation may not be completed. Therefore, ventricular function is affected in patients born preterm [5]. Factors influencing cardiac function in preterm infants have been less studied, and few studies have focused on breast milk. It has been observed that preterm infants fed with breast milk have better ventricular volume and systolic function, lower pulmonary vascular resistance, and affected strain and volumes compared to those fed with formula milk [6,7]. Although various studies in the literature have addressed the increased risk of cardiovascular disease in preterm infants and the role of breast milk, the relationship between prematurity-related issues and cardiovascular disease has not been adequately evaluated.

Previous studies have primarily focused on systemic hypertension and bronchopulmonary dysplasia-pulmonary hypertension, often comparing these conditions term birth individuals. However, data on the cardiovascular effects of accompanying neonatal factors are more limited. Our aim in this study is to conduct a detailed echocardiographic analysis of school-age children with a history of preterm birth, assessing cardiac function and exploring the relationship between echocardiographic parameters and neonatal factors.

## 2. Methods

This study included preterm infants who were born at or before 37 weeks of gestation and had a history of neonatal intensive care unit (NICU) hospitalization at Eskisehir Osmangazi University Hospital, a tertiary care hospital, between 2013 and 2017. Participants were invited for a follow-up visit in 2023–2024. Approval was obtained from the Eskişehir Osmangazi University Faculty of Medicine Non-Interventional Clinical Research Ethics Committee with the decision number 38 dated 21 March 2023. The study included children who were followed up in the neonatal unit and those who had no major congenital anomalies, cardiac anomalies, renal disease and poor echocardiographic imaging. Term birth children aged 7–11 years with non-specific chest pain and innocent murmurs were included as the control group. All controls had a gestational age of >37 weeks, with no history of chronic disease or hospitalization. On physical examination, there were no murmurs other than an innocent murmur, and the electrocardiogram was normal. No cardiac pathology was detected on echocardiography. All participants and parents provided written informed consent before participating in the study. Data collection included a detailed medical history, physical examination, and echocardiographic evaluation. Blood pressure values were obtained from the right arm at heart level with a properly sized cuff. The measurements were obtained while the child was sitting with a Nihon Kohden Life Scope N OPV-1500K (Nihon Kohden Corp. Shinjuku-ku, Tokyo, Japan), using the oscillometric method. The mean value was calculated from three consecutive measurements. 

### 2.1. Echocardiographic Evaluation

Echocardiographic assessment was performed using the Philips Epiq 3D device (Eindhoven, The Netherlands) with electrocardiography electrodes placed, and measurements were taken by an experienced pediatric cardiologist using a 5S probe. The echocardiographic examination utilized M-mode, 2D, Pulse Wave, and Tissue Doppler methods. The average of three consecutive measurements was taken for each parameter. Echocardiographic measurements were recorded according to the Pediatric Echocardiography Guidelines (American Society of Echocardiography Pediatric and Congenital Heart Disease Council) [8]. Measurements were performed in a double-blind manner from recorded image files.

Interventricular septum diameter (IVSD), left ventricular end-diastolic posterior wall diameter (LVPWD), left ventricular end-diastolic diameter (LVEDD), left ventricular end-systolic diameter (LVESD), and left ventricular fractional shortening (LVFS) were measured using M-mode from the parasternal short-axis window. Left ventricular ejection fraction (LVEF) was measured using the Simpson method from the apical four-chamber view. Left ventricular end-diastolic volume (LVEDV) and left ventricular end-systolic volume (LVESV) were measured from the apical four-chamber view.

Using with Pulsed wave Doppler, the velocities of mitral valve E and A waves were measured, and with Tissue Doppler, a’, e’, and s’ waves from the lateral and medial mitral annulus, isovolumetric contraction time (IVCT), systole, and isovolumetric relaxation time (IVRT) were assessed. The time interval from the peak to the end of the E wave was measured for mitral valve deceleration time. The duration of the A wave was measured from the beginning to the end. Mitral annular plane systolic excursion (MAPSE) was measured using M-mode from the lateral mitral annulus in the four-chamber view.

For right ventricular systolic function assessment, tricuspid annular plane systolic excursion (TAPSE) was measured using M-mode from the tricuspid valve lateral annulus in the apical four-chamber view. In the apical four-chamber view, pulse wave Doppler was used to record tricuspid valve E and A wave velocities, and Tissue Doppler recorded a’, e’, and s’ velocities from the lateral tricuspid annulus. RVEDA and RVESA calculations were performed to obtain right ventricular FAC. Myocardial Performance Index (MPI) was calculated using the formula MPI = (IVCT + IVRT)/EF.

For left ventricular longitudinal strain analysis, parasternal apical 3-chamber, 4-chamber, and 2-chamber views were obtained with Herz set to >60. For circumferential strain, basal, mid-ventricular, and apical short-axis views were acquired. For right ventricular strain, modified apical 4-chamber views including the right ventricular free wall and apex were recorded. Left atrium strain was recorded from the apical 4-chamber view. All strain analyses were performed offline in a double-blind manner by the same researcher using Qlab15.5.

### 2.2. Statistical Analysis

Data were analyzed using SPSS software version 23.0 (IBM Corp., Armonk, NY, USA). Continuous variables were expressed as mean ± standard deviation, and categorical variables were presented as frequencies and percentages. Comparisons between groups were made using the Student’s t-test for continuous variables and the chi-square test for categorical variables. Correlations between echocardiographic parameters and neonatal factors were evaluated using Pearson’s correlation coefficient. A *p*-value of <0.05 was considered statistically significant.

## 3. Results

The study included 32 children aged 7–11 years with a history of premature birth, and a control group of 32 term birth children matched for age and gender. The children with a history of premature birth and the term birth control group were similar in terms of age, gender, height, and body weight. The average birth weight in the premature group was 1479 ± 595 gr, while in the control group it was 3376 ± 157 gr, showing a statistically significant difference (*p* < 0.001). The average gestational age at birth in the premature group was 30.06 ± 2.80 weeks, compared to 38.41 ± 0.91 weeks in the term birth control group (*p* < 0.001). Systolic blood pressure was significantly lower in the premature group (108.10 ± 9.34 mmHg) compared to the control group (116.56 ± 6.53 mmHg) (*p* = 0.001). The demographic and blood pressure data of the premature and term birth groups are presented in Table 1.

Electronic records showed that 21 (65.6%) children in the premature group received surfactant therapy, and 16 (50%) of them received two or more doses. Additionally, the medical records indicated that 11 (34.4%) had a history of patent ductus arteriosus (PDA), 4 (12.5%) had pulmonary hypertension, 11 (34.4%) had bronchopulmonary dysplasia (BPD), 5 (15.6%) had necrotizing enterocolitis (NEC), 16 (50%) had retinopathy of prematurity (ROP), 9 (28.1%) had intraventricular hemorrhage (IVH), and 18 (56.3%) had required inotropic support. The average hospital stay for the premature group was 43.66 ± 37.15 days, with an average oxygen requirement of 22.34 ± 18.27 days. It was also found that 12 (37.5%) required nasal continuous airway pressure (NCPAP) and 20 (62.5%) required NCPAP plus intubation. None of the patients were receiving or being followed up for medical treatment due to conditions such as pulmonary hypertension or BPD. No cardiac symptoms were reported upon symptom inquiry.

### Comparison of Echocardiographic Data by Groups

The comparison of echocardiographic data between the premature and term birth groups is presented in Table 2 and Table 3.

In the premature group, Mitral E, A, tissue Doppler septal E, and tricuspid A values were significantly higher compared to the term birth group. MAPSE, IVRT, MPI, and LVESV were found to be lower. Strain echocardiography showed that LVGLS, RVFWS, and RVGLS were significantly lower in the premature group.

In the correlation analysis, a significant negative correlation was found between the duration of oxygen therapy and LVEDD (r = −0.407), LVESD (r = −0.430), and LVESV (r = −0.390) values in the premature group (*p* < 0.05). Similarly, a significant negative correlation was observed between the duration of NICU stay and LVEDD (r = −0.552), LVESD (r = −0.491), LVEDV (r = −0.505), and LVESV (r = −0.439) values in the premature group (*p* < 0.05). Gestational age was moderately negatively correlated with mitral E (r = −0.424) and A (r = −0.332) velocities. Positive correlations were found with IVRT (r = 0.381), MPI (r = 0.344), MAPSE (r = 0.337), IVSd (r = 0.303), and LVEDd (r = 0.280). A negative correlation was also found between gestational age and LVESV (r = −0.350), LV global longitudinal strain (r = −0.389), and RV free wall strain (r = −0.464) (Table 4).

## 4. Discussion

In the echocardiographic examination of school-aged children, while no significant pathology was detected using conventional echocardiographic methods, advanced echocardiographic techniques such as strain imaging revealed cardiac effects compared to term birth peers. In our study, children in the preterm group had significantly lower LV global longitudinal peak strain, RV free wall, RV 4-chamber strain, IVRT, MPI, MAPSE, and LVESV values compared to the control group (*p* < 0.05).

Recent research on preterm adolescents and young adults has shown an increased risk of hypertension, signs of vascular dysfunction, and significant changes in the heart [9,10,11,12]. A large study conducted in Sweden with 18-year-old males conscripted into military service found that the likelihood of systolic hypertension increased as gestational age fell below 32 weeks, with a nearly two-fold increase among those born at 24 weeks, the earliest gestational age in the study [13]. Another study conducted in Sweden from 1990 to 2007 with 5232 young adult women showed a progressive decrease in systolic and diastolic blood pressure with increasing gestational age [14]. In our study, the systolic blood pressure of the preterm group (108.10 ± 9.34 mmHg) was found to be lower compared to the control group (116.56 ± 6.53 mmHg), with no significant difference in diastolic blood pressure. Most studies in the literature have been conducted on adults and have found a relationship between preterm birth and hypertension. In contrast, our study, which involves a school-aged population, found lower systolic blood pressure. Although the control group consisted of healthy children, they were selected from patients visiting the hospital for benign murmurs and non-specific chest pain, which could influence blood pressure values. The preterm group consisted of patients born in our hospital and followed up there, with measurements made during routine follow-ups, which complicates standardization. The lack of ambulatory blood pressure monitoring in our study may also contribute to this discrepancy. Studies similar to ours have found no hypertension in school-aged children. For example, a study by Cheung et al. with preterm children with a median age of 8.2 years did not find hypertension in the preterm group without intrauterine growth restriction [15].

The literature highlights significant cardiac differences between preterm and term-born infants: (1) smaller left ventricle size and (2) diastolic filling pattern, with preterm children showing stiffer left ventricle walls compared to term children [16]. A study by Mohlkert et al., comparing 176 preterm children aged 6.5 years with 134 controls, found MAPSE 7% lower, lateral ventricular systolic myocardial velocity 11% lower, and fractional shortening 6% higher in the preterm group. There were no differences in myocardial performance index and systolic myocardial velocity of the septal wall between groups. Tissue Doppler echocardiography revealed lower myocardial velocities in the lateral wall of the left ventricle. The study suggested that even preterm children without hypertension showed deviations in systolic and diastolic function echocardiographically, highlighting the need for more extensive studies linking these findings to maternal and neonatal factors [17]. Similarly, in our study, although there was no significant difference in tissue Doppler imaging for mitral and tricuspid lateral wall myocardial velocities, septal E was significantly higher in the preterm group and negatively correlated with gestational age. MAPSE, IVRT, and MPI were lower in the preterm group compared to the control group and positively correlated with gestational age. Our study demonstrates that preterm children in this age group also experience impacts on systolic and diastolic functions and are related to gestational age. Unlike, Schubert et al., in a study with 87 children with a history of very low weight preterm birth and 29 term-born children aged 5–6 years, found no significant differences in MPI [18]. This study involved a similar age group but with a different patient population. In the study conducted by Flahault et al., involving young adults, it was shown that there were no differences in the E/A ratio and E/e’ ratio in mitral valve Doppler and mitral lateral tissue Doppler imaging. However, the e’ wave, representing early diastolic filling, was significantly lower in the premature group, similar to the LV s’ wave [19]. The group included in this study is of an older age group compared to our premature group, and it is thought that cardiac functions progressively deteriorate with age.

In a meta-analysis encompassing studies from neonatal period to young adulthood, 3136 participants (preterm = 1471; term = 1665) were evaluated. For young adults born before the 32nd week of gestation, differences were found in LVEDd, LVEDVI, and LV length compared to term controls. Preterm groups showed smaller LVEDD and LVEDV indices and shorter LV length throughout developmental stages (4). Flahault et al., evaluating 86 preterm-born and 85 term-born young adults aged 18–29 years, found similar left ventricular systolic function between term and preterm groups but smaller LV dimensions in the preterm group [19]. Our study aligns with the literature, showing that LV end-systolic volume and LVEDd are lower compared to term controls (though not statistically significant) and positively correlated with gestational age. Our findings were similar in terms of EF and FS, which were comparable between the preterm and control groups, though LVESV was lower.

A study by Mohlkert et al. comparing 176 preterm children aged 6.5 years with 134 controls found that TAPSE was lower in preterm children compared to term children, but no significant differences were found in right ventricular velocities or septal measurements [20]. Our study similarly evaluated a comparable age group and found TAPSE and FAC lower, and RVEDV and RVESV higher in the preterm group, although these differences were not statistically significant.

Our study also included analysis of left and right ventricular strain, showing lower LV global longitudinal and RV free wall and 4-chamber strain measurements compared to the control group. The EXPRESS (Extremely Preterm Infants in Sweden Study) followed 494 preterm children born before the 27th week of gestation for approximately 6.5 years and compared them with term controls. No differences in LV global longitudinal systolic strain or LV strain rate were observed between groups [16]. Similar to our findings, studies using cardiac magnetic resonance imaging on preterm birth young adults have also observed reductions in LV longitudinal strain and strain rates [10]. Previous research on infants and adults has shown that preterm birth children have lower strain, strain rate, and myocardial velocity compared to their peers [16]. While there are varied results in studies of similar age groups, our findings on reduced LV global longitudinal strain are consistent with studies of young adults.

Lewandowski et al. evaluated 102 preterm and 102 term young adults and 30 term adults, finding that RV peak diastolic strain was lower in preterm young adults compared to term young adults and adults, consistent with our findings [9]. Greer et al. compared 229 preterm adults with 100 term adults aged 26–30 years and found lower RV strain in preterm adults, suggesting that RV strain measurements may be associated with gestational age [21]. While these studies often involve older age groups, they also found lower RV strain in preterm individuals. This suggests that right ventricular strain abnormalities may begin in childhood. Additionally, similar to previous studies, Schubert et al. found RV strain correlated with gestational age [18].

There are limited studies on atrial strain measurements, especially in childhood. In our study, no significant difference was found in left atrial strain between the preterm and control groups. Limited studies on atrial strain in adults with a history of preterm birth have found lower atrial strain in preterm adults [22]. A study by Schuermans et al. evaluating atrial strain using cardiac magnetic resonance in young adults found higher LA booster strain in the preterm group, suggesting a compensatory mechanism [23]. As there are no similar studies on left atrial strain in children, comparisons are not possible. It is possible that atrial strain abnormalities may become apparent in adulthood, following ventricular diastolic dysfunction, but more extensive studies are needed.

Correlation analysis of neonatal factors revealed a negative and significant relationship between LVEDd (r = −0.407), LVESd (r = −0.430), LVESV (r = −0.390) and O_2_ requirement (*p* < 0.05). There was also a significant negative correlation between LVEDd (r = −0.552), LVESD (r = −0.491), LVEDV (r = −0.505), and LVESV (r = −0.439) with NICU stay duration (*p* < 0.05). Our study demonstrates that left ventricular dimensions and volumes are more affected as oxygen needs and NICU stay duration increase. This could be related to left and right ventricular interaction and chronic lung disease.

Limitations of our study include the small number of preterm children, lack of assessment of maternal factors (e.g., hypertension, diabetes, smoking, preeclampsia), unexamined comorbidities like hyperlipidemia, single-time point evaluation, absence of cardiac magnetic resonance imaging, and lack of evaluation of the relationship between breast milk and echocardiographic parameters. Additionally, the non-homogeneous distribution of numerical data and inability to grade factors like BPD were other limitations. Despite these, our study’s strengths include the evaluation of RV and LA strain analysis and its relationship with neonatal factors, which are rarely studied in children.

In conclusion, while conventional measurements in school-aged children with a history of preterm birth showed similar findings to term birth children, advanced echocardiographic examinations revealed impacts on systolic and diastolic functions of both ventricles. Regular echocardiographic monitoring of children with a history of preterm birth should be considered for routine practice. Further randomized prospective multicenter studies with larger patient groups are needed to evaluate the effects of neonatal factors on cardiac function and clinical implications. Given the potential impact of structural and physiological changes on cardiovascular disease and increased mortality in preterm individuals, periodic echocardiographic monitoring of cardiac function is important.

## Figures and Tables

**Table 1:** Demographic characteristics of the premature and term birth control groups.

	Premature Group (n = 32) Mean ± SD	Control Group (n = 32) Mean ± SD	*p*
Age (months)	115.25 ± 13.58	112.41 ± 15.75	0.441
Height (cm)	131.13 ± 11.06	135.12 ± 10.03	0.135
Weight (kg)	31.03 ± 8.93 kg	30.68 ± 9.03	0.879
Gender (F-M) (n-%)	14–18 (43.8–56.2)	16–16 (50–50)	0.802
Birth weight (g)	1479.06 ± 595.58	3375.94 ± 156.84	0.001
Gestational age (weeks)	30.06 ± 2.80	38.41 ± 0.91	0.001
Systolic blood pressure (mmHg)	108.10 ± 9.34	116.56 ± 6.53	0.001
Diastolic blood pressure (mmHg)	65.00 ± 8.05	67.19 ± 7.61	0.272
Heart rate (beats/min)	90.55 ± 10.65	88.91 ± 8.43	0.499

**Table 2:** Comparison of echocardiographic data by Gro.

Echocardiography Data	Premature Group (n = 32)	Control Group (n = 32)	*p*
Age (months)	115 ± 13	112 ± 16	0.671
Height (cm)	131 ± 11	135 ± 10	0.317
Weight (kg)	31 ± 9	31 ± 9	0.751
Mitral E (cm/s)	100.54 ± 17.99	86.13 ± 12.80	0.001
Mitral A (cm/s)	58.71 ± 12.38	51.25 ± 10.06	0.010
MV deceleration time * (ms)	127 (67–342)	146 (60–204)	0.545
Mitral A time (ms)	140.69 ± 4.91	140.72 ± 34.09	0.154
Mitral lateral E	19.35 ± 4.91	17.42 ± 3.04	0.064
Mitral lateral A	7.72 ± 2.55	6.79 ± 1.55	0.086
Mitral lateral S	10.59 ± 2.04	10.34 ± 2.20	0.634
Septal E	13.66 ± 2.05	12.22 ± 2.08	0.007
Septal A *	5.65 (3.45–39.20)	5.41 (3.80–8.94)	0.519
Septal S *	7.66 (4.41–9.12)	7.27 (5.28–8.61)	0.256
IVCT (ms)	53.31 ± 13.75	56.34 ± 12.93	0.367
Ejection time (ms)	268.47 ± 19.86	258.38 ± 28.07	0.102
IVRT(ms)	47.31 ± 11.96	57.47 ± 14.43	0.003
MPI	0.38 ± 0.07	0.45 ± 0.12	0.005
MAPSE (mm)	11.31 ± 2.03	12.32 ± 1.71	0.036
Tricuspid E	70.44 ± 18.81	67.30 ± 13.24	0.443
Tricuspid A	49.72 ± 18.81	43.46 ± 43.46	0.021
TR velosity	1.44 ± 0.59	1.27 ± 0.44	0.210
Tricuspid lateral E	14.47 ± 2.99	14.38 ± 2.60	0.907
Tricuspid lateral A	9.55 ± 2.98	9.20 ± 3.15	0.652
Tricuspid lateral S	12.44 ± 1.54	11.98 ± 2.69	0.272
TAPSE (mm)	16.53 ± 2.73	17.37 ± 2.69	0.215
IVSd * (mm)	5.97 (4.86–8.32)	6.65 (4.35–8.80)	0.096
LVEDd (mm)	37.84 ± 3.92	39.27 ± 4.06	0.157
LVPWd (mm)	6.41 ± 0.99	6.56 ± 0.92	0.535
LVESd (mm)	23.22 ± 3.11	24.05 ± 3.22	0.300
LVEF (%)	69.92 ± 5.60	69.29 ± 5.55	0.646
LVFS * (%)	37.55 (29.9–49.2)	38.5 (30–52.20)	0.809
LVEDV (mL)	63.00 ± 15.05	68.30 ± 15.84	0.175
LVESV (mL)	18.84 ± 6.40	22.59 ± 6.25	0.021
RVEDA (cm^2^)	9.00 ± 2.47	8.89 ± 2.36	0.858
RVESA (cm^2^)	5.61 ± 1.51	5.25 ± 1.45	0.334
RVFAC (%)	0.37 ± 0.10	0.39 ± 0.08	0.160

* non-parametric distribution.

**Table 3:** Strain echocardiography data.

Echocardiographic Data	Premature Group (n = 32)	Control Group (n = 32)	*p*
LV GLS (%)	−21.23 ± 1.88	−22.70 ± 1.87	0.003
LV CS (%)	−24.87 ± 3.48	−24.76 ± 2.95	0.896
RV FWS (%)	−21.99 ± 7.04	−30.54 ± 4.86	0.001
RV4Cs (%)	−19.74 ± 5.61	−26.12 ± 4.11	0.001
LA longitudinal strain peak (ms) *	342.5 (269–524) *	341 (186–476) *	0.989
LASr ED (%)	42.34 ± 11.51	43.38 ± 9.63	0.696
LAScd ED (%)	−34.42 ± 9.35	−33.89 ± 9.49	0.821
LASct ED (%)	−7.94 ± 6.87	−9.36 ± 7.13	0.422

* Median (Min–Max).

**Table 4:** Relationship between echocardiographic data and O_2_ requirement, NICU stay, and surfactant administration.

Echocardiographic Data	O_2_ Requirement	NICU Stay Duration	Surfactant Treatment	Gestational Age
	r	r	r	r
Mitral E (cm/s)	−0.201	−0.266	−0.031	−0.424
Mitral A (cm/s)	−0.133	−0.229	0.127	−0.332
MV deceleration time (ms)	−0.127	−0.223	0.172	0.045
Mitral A time (ms)	−0.191	−0.237	0.165	−0.075
Mitral lateral E	−0.002	0.082	−0.368	−0.217
Mitral lateral A	−0.270	−0.240	−0.033	−0.092
Mitral lateral S	−0.295	−0.277	−0.146	0.030
Septal E	−0.163	−0.110	0.180	−0.263
Septal A	−0.288	−0.270	0.298	−0.108
Septal S	−0.229	−0.171	0.003	−0.076
IVCT (ms)	0.105	0.021	−0.088	−0.103
Sistol (ms)	−0.078	−0.231	0.074	−0.182
IVRT(ms)	0.044	−0.138	−0.074	0.381
MPI	0.145	0.022	−0.147	0.344
MAPSE (mm)	−0.291	−0.291	0.069	0.373
Tricuspid E	−0.133	−0.196	−0.019	−0.040
Tricuspid A	−0.163	0.066	0.130	−0.249
TR velosity	0.055	−0.049	0.062	−0.111
Tricuspid lateral E	0.156	0.140	0.211	0.003
Tricuspid lateral A	−0.199	−0.52	−0.130	−0.101
Tricuspid lateral S	0.030	0.165	−0.022	−0.127
TAPSE (mm)	−0.009	−0.118	0.196	0.144
IVSd (mm)	−0.288	−0.308	0.073	0.303
LVEDd (mm)	−0.407	−0.552	−0.056	0.280
LVPWd (mm)	−0.300	−0.272	−0.261	0.128
LVESd (mm)	−0.430	−0.491	−0.062	0.226
LVEF (%)	0.133	0.054	−0.003	−0.061
LVFS (%)	0.071	0.173	−0.097	−0.046
LVEDV (mL)	−0.330	−0.505	−0.189	0.236
LVESV (mL)	−0.390	−0.439	−0.076	−0.350
RVEDA (cm^2^)	−0.139	−0.185	0.217	0.091
RVESA (cm^2^)	−0.197	−0.229	0.146	0.012
RVFAC (%)	0.111	0.083	0.082	0.215
LV GLS (%)	0.173	0.184	−0.081	0.389
LV CS (%)	0.057	0.106	−0.155	0.039
RV FWS (%)	−0.075	0.063	0.073	0.464
RV4Cs (%)	−0.079	−0.027	−0.026	−0.428
LA longitudinal strain peak (ms)	−0.008	−0.092	−0.120	0.096
LASr ED (%)	0.128	0.077	−0.220	0.048
LAScd ED (%)	−0.113	−0.080	0.280	0.010
LASct ED (%)	−0.060	−0.020	−0.023	−0.075

*p* < 0.05. MV: mitral valve, IVCT: isovolumetric contraction time, IVRT: isovolumetric relaxation time, MPI: myocardial performance index, MAPSE: mitral annular plane systolic excursion, TR: tricuspid regurgitation, RV: right ventricle, TAPSE: tricuspid annular plane systolic excursion, IVSd: interventricular septum diameter, LVEDd: left ventricular end-diastolic diameter, LVPWd: left ventricular posterior wall end-diastolic diameter, LVESd: left ventricular end-systolic diameter, EF: ejection fraction, FS: fractional shortening, LVEDV: left ventricular end-diastolic volume, LVESV: left ventricular end-systolic volume, LV: left ventricle, FAC: fractional area change, RVEDA: right ventricular end-diastolic area, RVESA: right ventricular end-systolic area, LVGLS: left ventricular global longitudinal strain, LVCS: left ventricular circumferential strain, RV FWS: right ventricular free wall strain, RV4Cs: right ventricular 4-chamber strain, LASr ED: left atrium reservoir strain, LAScd ED: left atrium conduit strain, LASct ED: left atrium contraction strain.

## Data Availability

No new data were created or analyzed in this study. Data sharing is not applicable to this article.
